# Supplementary figures and images for: Association between inflammatory cytokines and immune–checkpoint molecule in rheumatoid arthritis
Source: PLoS One. 2021 Nov 18;16(11):e0260254. doi: 10.1371/journal.pone.0260254 (PMC8601500; doi:10.1371/journal.pone.0260254)

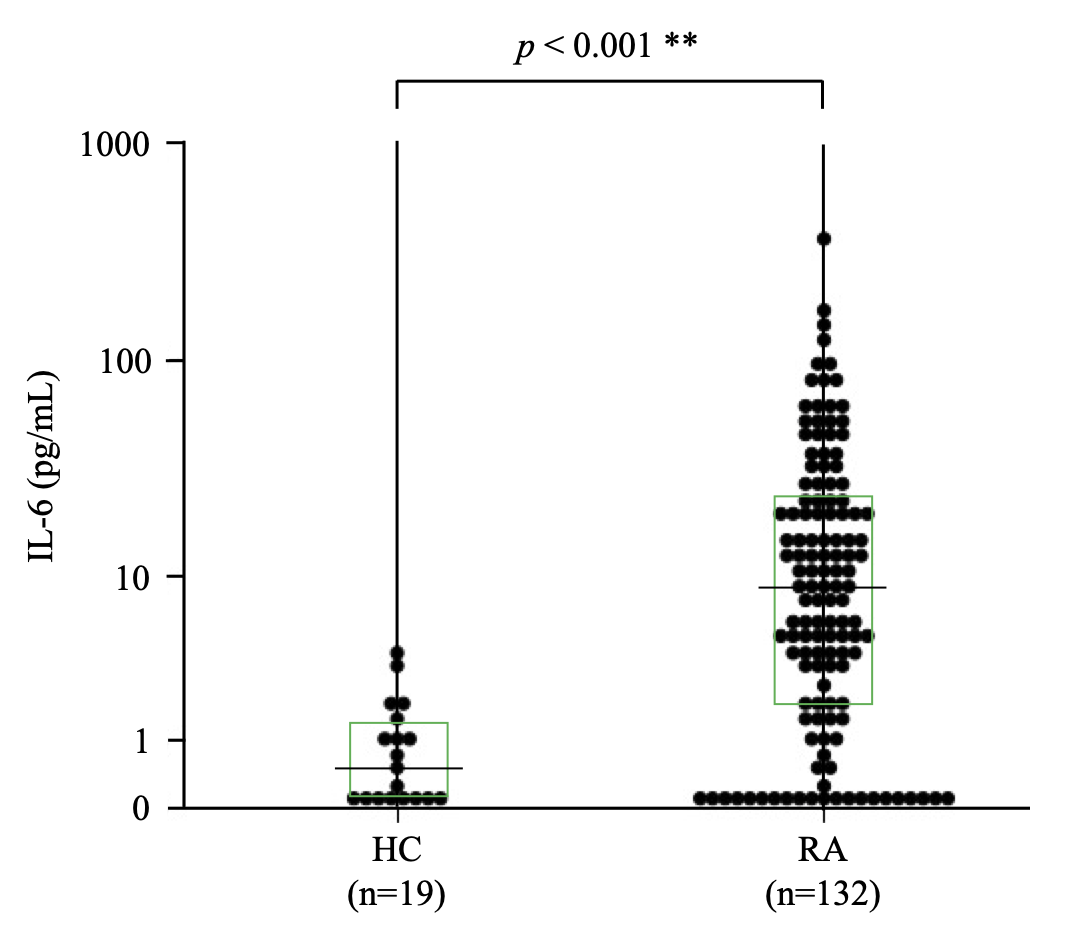

Supplement: S1 Fig — Serum levels of IL-6 in RA patients were significantly higher compared to those in healthy HCs. Statistical significance was determined by Mann-Whitney U test. (TIFF) [file pone.0260254.s001.tiff]

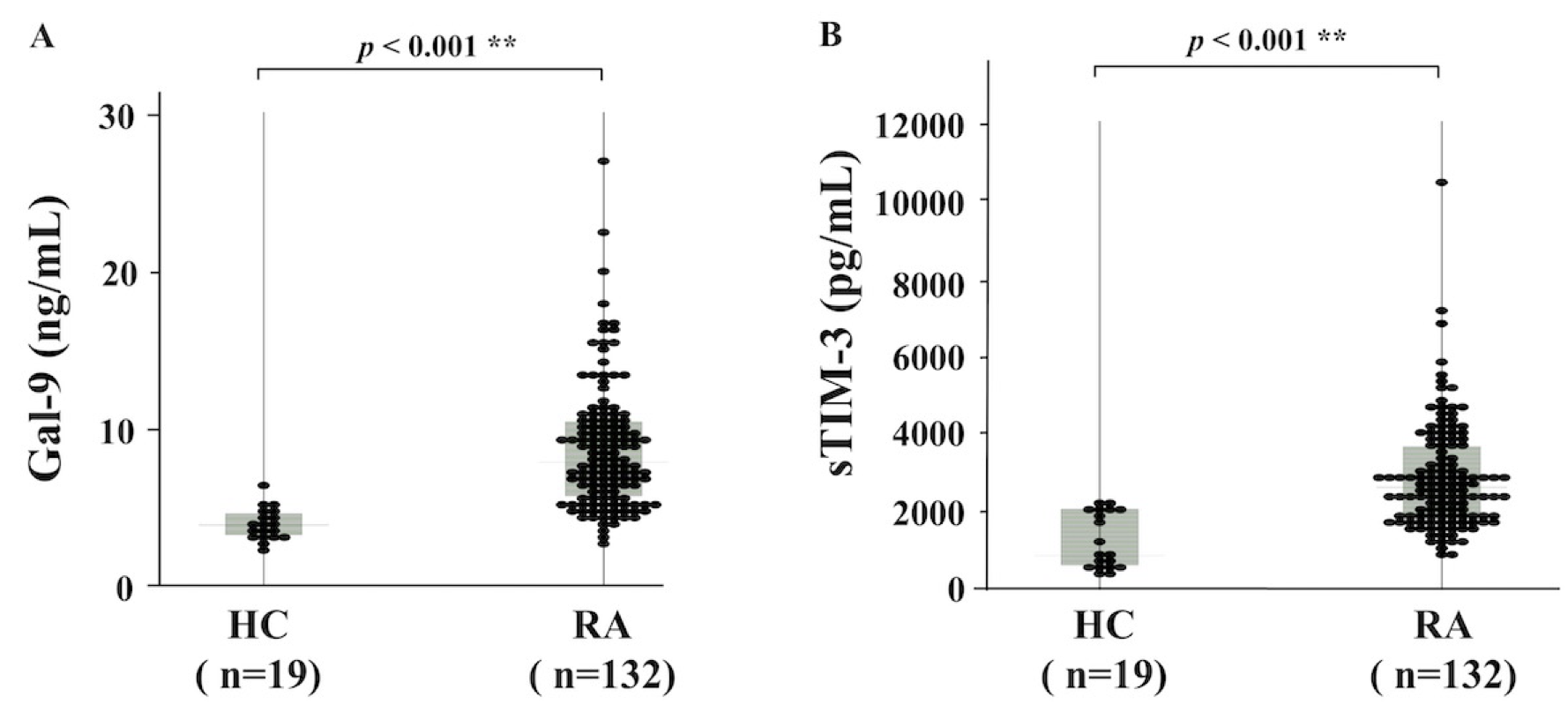

Supplement: S2 Fig — (A) Serum levels of Gal-9 in RA patients were significantly higher compared to those in HCs. (B) Serum levels of sTIM-3 in RA patients were significantly higher compared to those in HCs. Statistical significance was determined by Mann-Whitney U test. (TIFF) [file pone.0260254.s002.tiff]

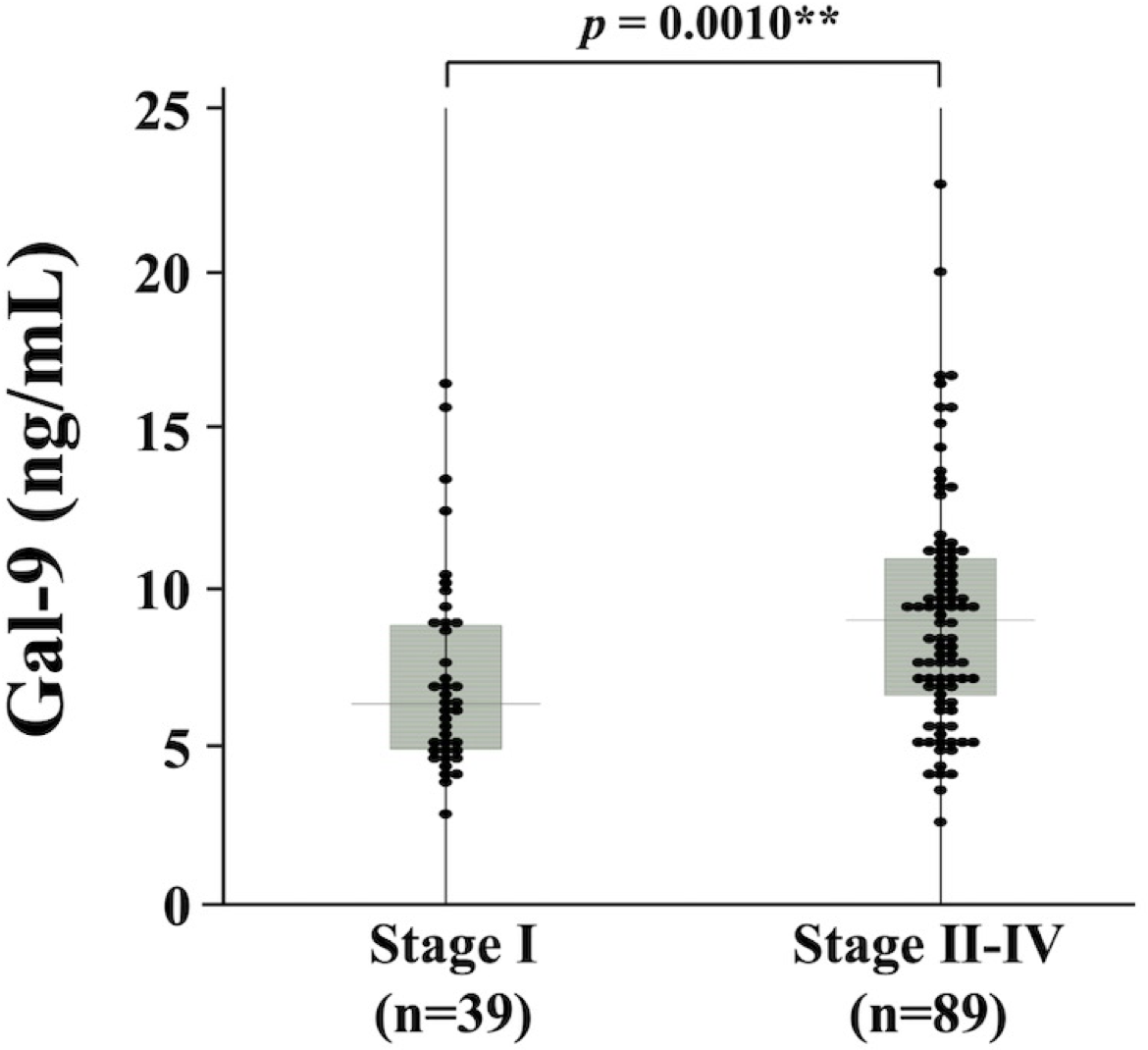

Supplement: S3 Fig — Serum levels of Gal-9 were significantly higher in RA patients with advanced joint damage (stage II–IV) compared to those without advanced joint damage (Stage I). Statistical significance was determined by Mann-Whitney U test. (TIFF) [file pone.0260254.s003.tiff]

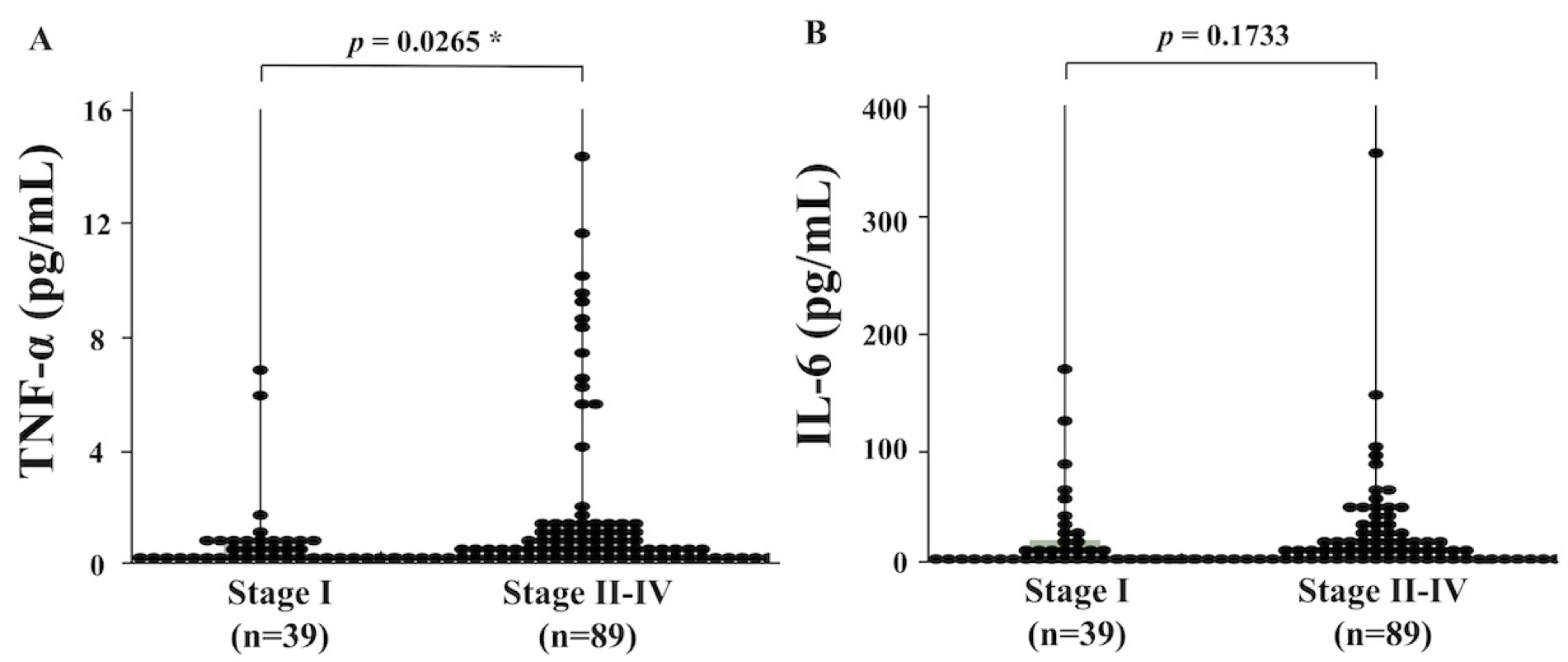

Supplement: S4 Fig — (A) Serum levels of TNF-α in RA patients with advanced joint damage were significantly higher than those in RA patients without advanced joint damage. (B) Serum levels of IL-6 in RA patients with advanced joint damage (Stage II-IV) were higher than those in RA patients without advanced joint damage (Stage I); however, there was no significant difference. Statistical significance was determined by Mann-Whitney U test. (TIFF) [file pone.0260254.s004.tiff]
