# Supplementary material for: Association between inflammatory cytokines and immune–checkpoint molecule in rheumatoid arthritis
Source: PLoS One. 2021 Nov 18;16(11):e0260254. doi: 10.1371/journal.pone.0260254 (PMC8601500; doi:10.1371/journal.pone.0260254)
Supplement: S1 File — (DOCX) [file pone.0260254.s005.docx]

**What data are required and what is meant by minimal data set? PLOS defines the “minimal data set” to consist of the data set used to reach the conclusions drawn in the manuscript with related metadata and methods, and any additional data required to replicate the reported study findings in their entirety. Authors do not need to submit their entire data set, or the raw data collected during an investigation. Please submit the following data.**

**・The values behind the means, standard deviations and other measures reported;**

**・The values used to build graphs;**

**・The points extracted from images for analysis.**

| **Fig. #** | **Median** | **IQR** | **Statistical method used** | **P value** | **# sample** |
| --- | --- | --- | --- | --- | --- |
| **Fig.1** |  |  | Spearman’s rank correlation  coefficient |  |  |
| IL-6 | 8.45 | 1.87-21.24 |  | p < 0.001* | 105 |
| TNF-α | 0.52 | 0.22-1.04 |  |  | 105 |
| **Fig. 2** |  |  | Spearman’s rank correlation  coefficient |  |  |
| ACPA<200 |  |  |  |  |  |
| IL-6 | 8.37 | 1.87-23.84 |  | p <0.001 | 82 |
| DAS28-ESR | 3.00 | 2.10-3.82 |  |  | 82 |
| TNF-α | 0.48 | 0.15-0.99 |  | p = 0.018 | 82 |
| DAS28-ESR | 3.00 | 2.10-3.82 |  |  | 82 |
| ACPA≧200 |  |  |  |  |  |
| IL-6 | 7.21 | 1.58-19.52 |  | p = 0.028 | 41 |
| DAS28-ESR | 2.88 | 20.05-3.75 |  |  | 41 |
| TNF-α | 0.48 | 0.16-0.96 |  | p = 0.018 | 41 |
| DAS28-ESR | 2.88 | 2005-3.75 |  |  | 41 |
| **Fig.3** |  |  | Spearman’s rank correlation  coefficient |  |  |
| ACPA<200 |  |  |  |  |  |
| IL-6 | 8.37 | 1.87-23.84 |  | p = 0.0024 | 89 |
| Gal-9 | 7.84 | 5.69-10.39 |  |  | 89 |
| TNF-α | 0.48 | 0.15-0.99 |  | p = 0.0016 | 89 |
| Gal-9 | 7.84 | 5.69-10.39 |  |  | 89 |
| ACPA≧200 |  |  |  |  |  |
| IL-6 | 7.21 | 1.58-19.52 |  | p = 0.0080 | 43 |
| Gal-9 | 7.60 | 5.57-10.34 |  |  | 43 |
| TNF-α | 0.48 | 0.16-0.96 |  | p= 0.0046 | 43 |
| Gal-9 | 7.60 | 5.57-10.34 |  |  | 43 |
| **Fig.4** |  |  | Spearman’s rank correlation  coefficient |  |  |
| ACPA<200 |  |  |  |  |  |
| IL-6 | 8.37 | 1.87-23.84 |  | p = 0.0050 | 86 |
| sTIM-3 | 2627.99 | 1882.71-3676.32 |  |  | 86 |
| TNF-α | 0.48 | 0.15-0.99 |  | p = 0.0034 | 86 |
| sTIM-3 | 2627.99 | 1882.71-3676.32 |  |  | 86 |
| ACPA≧200 |  |  |  |  |  |
| IL-6 | 7.21 | 1.58-19.52 |  | p = 0.1027 | 42 |
| sTIM-3 | 2618.47 | 1846.16-3668.95 |  |  | 42 |
| TNF-α | 0.48 | 0.16-0.96 |  | p = 0.6394 | 42 |
| sTIM-3 | 2618.47 | 1846.16-3668.95 |  |  | 42 |
| **Fig.5** |  |  | Spearman’s rank correlation  coefficient |  |  |
| Stage I |  |  |  |  |  |
| TNF-α | 0.48 | 0.15-0.99 |  | p = 0.113 | 39 |
| Gal-9 | 7.82 | 5.63-10.38 |  |  | 39 |
| IL-6 | 8.37 | 1.66-23.85 |  | p = 0.005 | 39 |
| Gal-9 | 7.82 | 5.63-10.38 |  |  | 39 |
| Stage II-IV |  |  |  |  |  |
| TNF-α | 0.48 | 0.16-0.98 |  | p = 0.001 | 89 |
| Gal-9 | 7.60 | 5.49-10.31 |  |  | 89 |
| IL-6 | 7.72 | 1.58-23.28 |  | p = 0.029 | 89 |
| Gal-9 | 7.60 | 5.49-10.31 |  |  | 89 |
| **Fig.6** |  |  | Spearman’s rank correlation  coefficient |  |  |
| Stage I |  |  |  |  |  |
| TNF-α | 0.31 | 0.072-0.76 |  | p = 0.625 | 16 |
| Gal-9 | 6.98 | 5.11-9.71 |  |  | 16 |
| IL-6 | 3.57 | 0.045-10.77 |  | p = 0.013 | 16 |
| Gal-9 | 6.98 | 5.11-9.71 |  |  | 16 |
| Stage II-IV |  |  |  |  |  |
| TNF-α | 0.475 | 0.15-0.95 |  | p = 0.004 | 26 |
| Gal-9 | 7.58 | 5.56-10.15 |  |  | 26 |
| IL-6 | 7.21 | 1.58-19.46 |  | p = 0.397 | 26 |
| Gal-9 | 7.58 | 5.56-10.15 |  |  | 26 |
